# Transcription of the mating-type-regulated lncRNA *IRT1* is governed by TORC1 and PKA

**DOI:** 10.1007/s00294-016-0639-6

**Published:** 2016-08-12

**Authors:** Fabien Moretto, Folkert J. van Werven

**Affiliations:** Cell Fate and Gene Regulation Laboratory, The Francis Crick Institute, 44 Lincoln’s Inn Fields, London, WC2A 3LY UK

**Keywords:** IME1, Sporulation, Gametogenesis, Long non-coding RNA, IRT1, Rme1, TORC1, Protein kinase A, Tup1, Mating type, Nutrients, Signalling, Transcription

## Abstract

Cell fate decisions are controlled by multiple cell-intrinsic and -extrinsic factors. In budding yeast, the decision to enter gametogenesis or sporulation is dictated by nutrient availability and mating type. Recently, we showed that in diploid cells harbouring opposite mating types (*MAT*a and *MAT*α), the protein kinase A (PKA) and target of rapamycin complex I (TORC1) signalling pathways integrate at the promoter of the master regulatory transcription factor *IME1* to control sporulation via nutrient availability (Weidberg, et al. 2016). In cells with a single mating type (*MAT*a or *MAT*α), however, *IME1* is repressed by transcription through the *IME1* promoter of a long non-coding RNA called *IRT1*, which prevents this cell type from undergoing sporulation. Here, we investigated the role of nutrient signalling in mating-type control of *IME1*. We find that expression of *IRT1*, like *IME1* itself, depends on nutrient availability and the activities of PKA and TORC1. *IRT1* transcription is repressed when nutrients are ample and TORC1 and PKA are active. In contrast, inhibition of PKA and TORC1 is sufficient to recruit Rme1 to the *IRT1* promoter and induce *IRT1*-mediated repression of *IME1*. Finally, we provide evidence that *IRT1* and *IME1* are co-repressed by the Tup1–Cyc8 complex when nutrients are ample. Thus, in cells with a single mating-type nutrient availability regulates mating-type repression of *IME1* and sporulation. Our results indicate that there is a hierarchy between nutrient and mating-type signals in controlling the decision to enter sporulation.

## Introduction

Cell fate decisions are regulated by a multitude of intrinsic and extrinsic signals. How multiple signals are integrated to make a binary cell fate choice is often not well understood.

Budding yeast gametogenesis or sporulation is an ideal model system to study this problem. When the signal requirements to enter sporulation are met, cells induce a gene expression cascade to generate four haploid spores from a diploid cell (Honigberg and Purnapatre 2003; Jin and Neiman 2016; Kassir et al. 2003; van Werven and Amon 2011; Vershon and Pierce 2000).

Nutrient and mating-type signals control the decision leading to meiosis and gamete or spore formation. These signals integrate at the promoter of the master regulator for entry into sporulation, called *IME1* (Kassir et al. 1988; Nachman et al. 2007). When nutrients are ample, *IME1* is repressed because the two major nutrient sensing signalling pathways, protein kinase A and target of rapamycin complex I (TORC1), are active (Weidberg et al. 2016). In the absence of nitrogen and glucose compounds in the growth medium, TORC1 and PKA activities are repressed and *IME1* is strongly induced.

The mating-type loci also control entry into sporulation. *IME1* can only be induced in diploid cells because the a1–α2 complex, which expression requires both mating loci (*MAT*a and *MAT*α), represses the transcription of *RME1* (Mitchell and Herskowitz 1986). Cells with a single mating type (*MAT*a or *MAT*α), however, exhibit high levels of Rme1 which in turn represses *IME1* via an unusual mechanism (Covitz and Mitchell 1993; van Werven et al. 2012). Rme1 activates the transcription of the long non-coding RNA *IRT1*, which is expressed from upstream in the *IME1* promoter and spans almost the complete promoter. Repression of *IME1* by *IRT1* transcription requires repressive chromatin marks such as histone H3 lysine 36 methylation and lysine 4 di-methylation (van Werven et al. 2012).

How nutrient availability regulates *IRT1* transcription is not well understood. Here, we demonstrate that *IRT1* transcription, like *IME1*, is under control of the TORC1 and PKA nutrient sensing pathways. In addition, we provide evidence that the transcriptional repressor Tup1–Cyc8, like *IME1,* represses *IRT1*. We propose that co-repression of *IME1* and *IRT1* serves as a fail-safe mechanism for mating-type control of sporulation. Overall, our results suggest that a hierarchy between nutrient and mating-type signals controls the decision to enter sporulation.

## Materials and methods

### Strains

SK1 strain background was used for the experiments throughout this manuscript. The *tup1*Δ strain was generated using one-step deletion protocol as described by Longtine et al. (1998). The *tpk1as* and Rme1-V5 alleles were described previously (van Werven et al. 2012; Weidberg et al. 2016). The genotypes of the strains used for this study are described in Table 1.

**Table 1:** Yeast strains

FW1509	*MATa, ho::LYS2, lys2, ura3, leu2::hisG, his3::hisG, trp1::hisG*
FW1760	*MATa ho::LYS2, lys2, ura3, leu2::hisG, his3::hisG, trp1::hisG, tpk1::tpk1M164G, tpk2::KanMX6, tpk3:: TRP1*
FW1765	*MAT𝛼 ho::LYS2, lys2, ura3, leu2::hisG, his3::hisG, trp1::hisG, tpk1::tpk1M164G, tpk2::KanMX6, tpk3:: TRP1, rme1::RME1*-*3xV5::HIS3*
FW2752	*MATa, ho::LYS2, lys2, ura3, leu2::hisG, his3::hisG, trp1::hisG, tup1:: KanMX6*

### Growth conditions

**Fig. 1:**
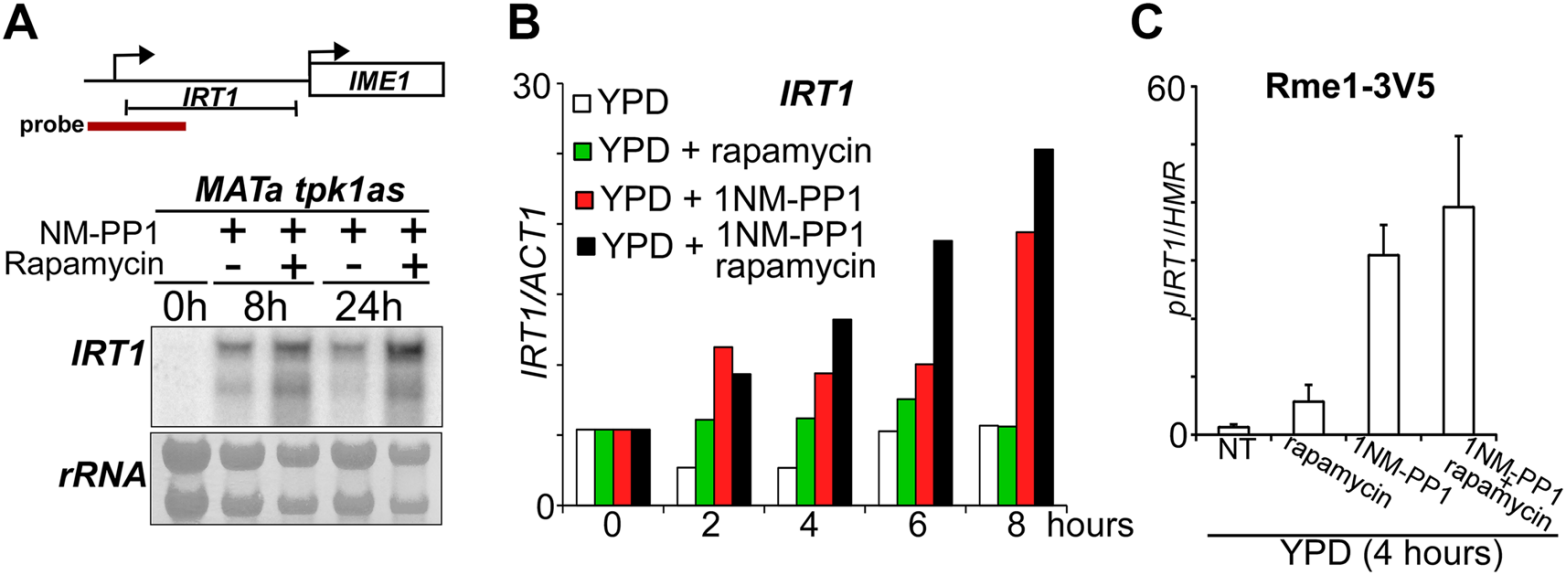
*IRT1* is induced in rich medium when PKA and TORC1 are inhibited. **a** Overview of *IRT1/IME1* locus (*top*), and northern blot of *IRT1* expression (*bottom*). Haploid cells harbouring the *tpk1M164G*, *tpk2*Δ, *tpk3*Δ alleles (*tpk1as*) (FW1760) were grown overnight in YPD, diluted into YPD plus 1NM-PP1 or rapamycin/1NM-PP1, and samples were taken at the indicated time points. Total RNA was isolated, separated by gel electrophoresis, blotted and probed for *IRT1.* **b** *IRT1* RNA quantification in haploid cells harbouring *tpk1as* (FW1760). Cells were grown overnight in YPD, diluted into fresh YPD in absence or presence of rapamycin, 1NM-PP1, or rapamycin/1NM-PP1. Samples were taken at the indicated time points. Total RNA was isolated, reverse transcribed, and *IRT1* mRNA levels were measured by quantitative PCR. Signals were normalized to *ACT1* levels. The average signals of two biological experiments are shown. **c** Cells were grown and treated as in **b**. Rme1 binding to the *IRT1* promoter was measured by chromatin immunoprecipitation at 4 h after treatment. Cells were fixed with formaldehyde, following immunoprecipitation of Rme1 tagged with 3xV5 epitope (FW1765) from chromatin extracts (see “Materials and methods” for details). The recovered DNA was quantified by real-time PCR with primers corresponding to the *IRT1* promoter (*pIRT1*). Signals were normalized to the silent mating-type locus (*HMR*), which does not bind Rme1

**Fig. 2:**
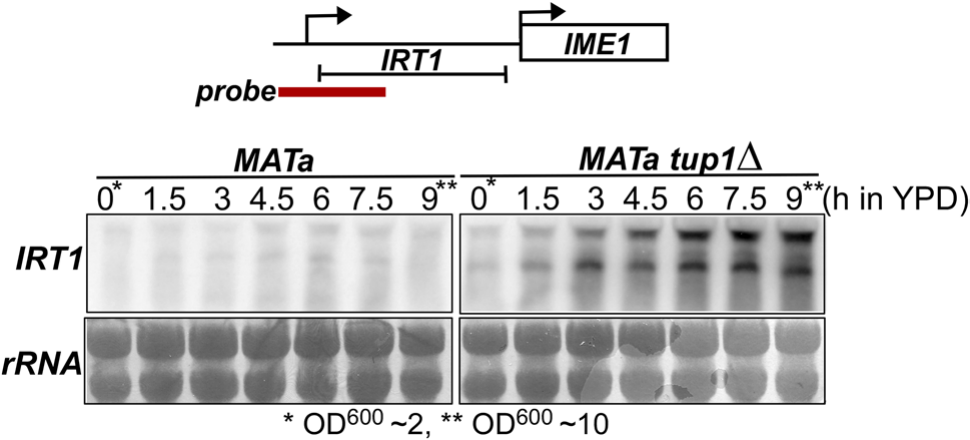
Northern blot analysis of *IRT1* in wild-type and *tup1*Δ cells during diauxic shift. Haploid control (FW1509) or *tup1*Δ (FW2752) cells were grown overnight, diluted in YPD (OD600 = 2) and samples were taken at the indicated time points. Total RNA was isolated, separated by gel electrophoresis, blotted and probed for *IRT1*

For Fig. 1, cells were grown overnight in YPD (1 % yeast extract, 2 % peptone, 2 % glucose) at 30 °C, then diluted to fresh YPD (OD600 = 1) and treated with different drugs. For Fig. 2, cells were grown overnight and diluted to OD600 = 2, and samples were taken at the indicated time points.

### Northern blot analysis

To measure *IRT1* expression by northern blot we used a northern blot protocol which has been described previously (Koster et al. 2014). The *IRT1* probe has been described previously (van Werven et al. 2012).

### RT-PCR

The RT-PCR protocol was described previously (Weidberg et al. 2016). In short, 750 ng of DNase-treated total RNA was used for the reverse transcription reaction, and single-stranded cDNA were quantified by real-time PCR using an SYBR green mix (Life Technologies) on a 7500 Fast Real-Time PCR system (Life Technologies). Signals were normalized to *ACT1* transcripts levels. The primer sequences are available on request.

### Chromatin immunoprecipitation

Chromatin immunoprecipitation experiments were executed as described previously (Weidberg et al. 2016). Cells were fixed with 1 % formaldehyde for 20 min, the reaction was quenched with 125 mM glycine. Cells were disrupted using a mini beadbeater (BioSpec), and crosslinked chromatin was sheered by sonication using the Bioruptor (Diagenode, 7 cycles of 30 s on/off). Chromatin extracts were then incubated with anti-V5 agarose beads (Sigma) for 2 h at room temperature, and beads were washed accordingly. To measure Rme1 binding, input and ChIP samples were quantified by real-time PCR using SYBR green mix (Life Technologies) and primers corresponding to the *IRT1* promoter on a 7500 Fast Real-Time PCR system (Life Technologies). The mating-type locus (HMR) was used as a non-binding negative control. Primer sequences are available on request.

## Results and discussion

### TORC1 and PKA control *IRT1* transcription

Previous work showed that the TORC1 and PKA nutrient sensing pathways control the promoter of *IME1*, the master regulator of entry into sporulation (Weidberg et al. 2016). The *IME1* promoter is not only regulated by nutrient availability, but also by mating type (Covitz and Mitchell 1993; Kassir et al. 1988; Mitchell and Herskowitz 1986). In cells with a single mating type, *IME1* is repressed by transcription of the long non-coding RNA called *IRT1*, which transcribes through the *IME1* promoter. Like *IME1*, induction of *IRT1* occurs when haploid cells are starved (van Werven et al. 2012). It is unclear, however, how *IRT1* stays repressed when nutrients are ample. We hypothesized that similar to *IME1*, *IRT1* is controlled by TORC1 and PKA signalling. To test this, we used the analogue sensitive allele of PKA (*tpk1as*) that was described previously and the TORC1 inhibitor rapamycin (Weidberg et al. 2016). When we grew cells in nutrient-rich conditions (YPD) and inhibited PKA using the small molecule 1NM-PP1, *IRT1* was strongly induced (Fig. 1a, b). Inhibition of TORC1 alone following rapamycin treatment had almost no effect on *IRT1* transcription, but when combined with inhibition of PKA *IRT1* levels increased further (Fig. 1a, b). We also measured the binding of Rme1, the activator of *IRT1*, to the *IRT1* promoter (van Werven et al. 2012). We found that inhibition of PKA alone led to strong recruitment of Rme1, whereas inhibition of TORC1 resulted in low levels of Rme1 recruitment. The combined repression of PKA and TORC1 had little added effect when compared to PKA alone suggesting that PKA is the main regulator of *IRT1* under these conditions. Similar observations were made for *IME1* because inhibition of PKA has a much stronger effect on *IME1* activation than inhibition of TORC1 (Weidberg et al. 2016). These results show that, like *IME1*, *IRT1* transcription is under the control of the PKA and TORC1 signalling pathways.

### Tup1 is required for *IRT1* repression

Having established that TORC1 and PKA control *IRT1* transcription in cells with a single mating type, we next examined whether *IRT1* and *IME1* also share the same repressor protein complex. In nutrient-rich conditions, the *IME1* promoter is repressed by Tup1–Cyc8 complex, which binds in the middle of the *IME1* promoter (between −800 and −1000 base pairs upstream to the transcription start site) (Mizuno et al. 1998; Weidberg et al. 2016). This transcriptional repressor interacts with sequence-specific transcription factors and histone de-acetyltransferases to repress promoters (Keleher et al. 1992; Tzamarias and Struhl 1994; Watson et al. 2000; Wong and Struhl 2011). Tup1–Cyc8 is evicted from the *IME1* promoter to de-repress *IME1*, following nutrient starvation and inhibition of PKA/TORC1 (Weidberg et al. 2016). To test whether *IRT1* is also regulated by Tup1–Cyc8, we measured *IRT1* expression in the wild-type and *tup1*Δ cells in nutrient-rich conditions and post-diauxic shift when nutrients were largely used from the growth medium. We found that *IRT1* was de-repressed in the *tup1*Δ cells exposed to high nutrients (OD600 ~ 2). Interestingly, the levels of *IRT1* were significantly higher when *tup1*Δ cells were grown to high density (OD600 ~ 10). We conclude that Tup1–Cyc8 contributes to *IRT1* repression.

Our result shows that, like *IME1*, *IRT1* (at least in part) is regulated by Tup1–Cyc8. How Tup1–Cyc8 represses both *IRT1* and *IME1* remains unclear. Promoter scanning showed that Tup1 binding peaked in the middle of the *IME1* promoter (Weidberg et al. 2016). We observed no difference in Tup1 binding between haploid and diploid cells (unpublished data). Given that the distance between the Rme1 and Tup1 binding in the *IME1* promoter is relatively short (under one kilobase), we propose that Tup1–Cyc8 establishes a repressive chromatin state at the *IME1* promoter that spreads to the *IRT1* promoter. Our observation that *IRT1* expression was further induced during post-diauxic shift in *tup1*Δ cells suggests that Tup1–Cyc8 is not the sole repressor of *IRT1* and other factors may also contribute. Another explanation is that the activator of *IRT1,* Rme1, is regulated by nutrient availability. It is known that Rme1 levels are cell cycle regulated, peaking during late M/early G1 (Toone et al. 1995). Upon post-diauxic shift a large fraction of cells arrest in G1, whereas in nutrient-rich conditions cells continuously progress through the cell cycle. More work is needed to fully depict how *IRT1* repression is controlled.

## Conclusions and model

**Fig. 3:**
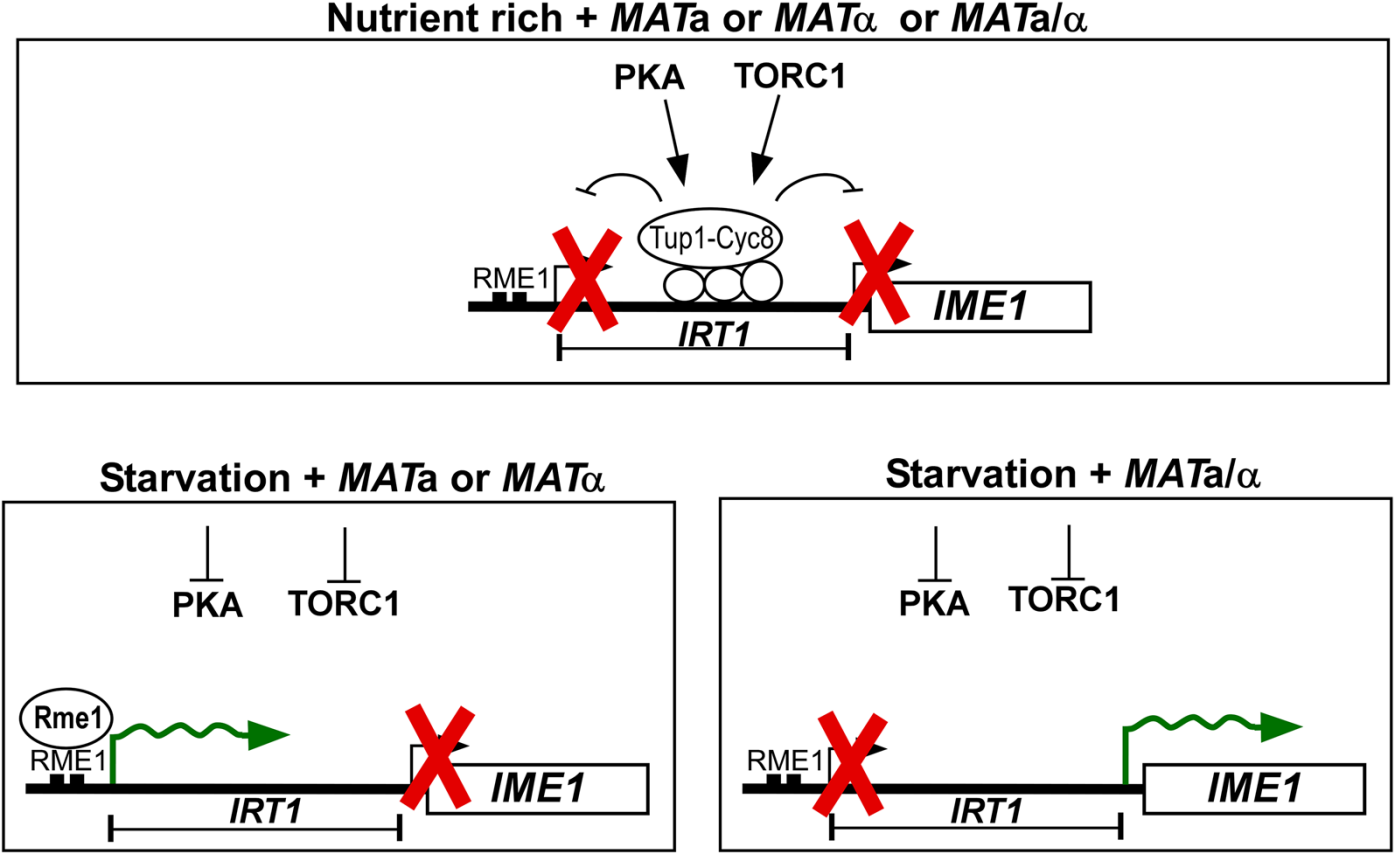
Model of nutrient and mating-type control of *IME1*. Under nutrient-rich conditions, TORC1/PKA repress *IME1* and *IRT1* via Tup1–Cyc8 in diploid/haploid cells harbouring either both or single mating types (*MAT*a/α, *MAT*a or *MAT*α). During starvation, cells with a single mating type (*MAT*a or *MAT*α) induce *IRT1*-mediated repression of *IME1*, whereas *MAT*a/α diploid cells induce *IME1* and enter sporulation

It has been known for decades that nutrient availability and mating type are key regulators of *IME1* and entry into sporulation. How nutrients regulate mating-type control of *IME1* was not understood. Here, we show that transcription of the mating-type-regulated lncRNA *IRT1* is under the control of the same nutrient sensing pathways (TORC1 and PKA) as *IME1*. In addition, we provide evidence that *IRT1* and *IME1* are under control of the same repressor complex, Tup1–Cyc8. These findings have several implications. First, they show that mating-type control of *IME1* is not active when nutrients are ample (Fig. 3). In other words, there is a hierarchy, in which nutrient repression is prevalent over mating-type control. Second, co-regulation of *IME1* and *IRT1* could serve as a fail-safe mechanism for mating-type control of *IME1*. Indeed, it has been known that mating-type control of sporulation is essential for preventing haploid cells to enter meiosis, which would be lethal as two consecutive cell divisions would attempt the segregation of a haploid genome into four spores. If *IRT1* and *IME1* were under control of different signalling pathways and different transcriptional repressors, this could have led to a mis-regulation of mating-type control of *IME1*. Our observation that *IME1* and *IRT1* expressions are regulated by the same nutrient sensing pathways and by the same transcriptional repressor complex ensures that *IRT1* is activated at the same time when the nutrient requirements for *IME1* are met and vice versa. Overall, we propose that the hierarchy between nutrient and mating-type signals ensures that diploid, and not haploid, cells induce *IME1* and enter sporulation.
